# Examination of the Abscission-Associated Transcriptomes for Soybean, Tomato, and Arabidopsis Highlights the Conserved Biosynthesis of an Extensible Extracellular Matrix and Boundary Layer

**DOI:** 10.3389/fpls.2015.01109

**Published:** 2015-12-15

**Authors:** Joonyup Kim, Srivignesh Sundaresan, Sonia Philosoph-Hadas, Ronghui Yang, Shimon Meir, Mark L. Tucker

**Affiliations:** ^1^Soybean Genomics and Improvement Lab, Agricultural Research Service, United States Department of AgricultureBeltsville, MD, USA; ^2^Department of Postharvest Science of Fresh Produce, Agricultural Research Organization, The Volcani CenterBet-Dagan, Israel; ^3^The Robert H. Smith Faculty of Agriculture, Food and Environment, The Hebrew University of JerusalemRehovot, Israel

**Keywords:** abscission, Arabidopsis, ethylene, *IDA*, soybean, tomato, transcriptome, cuticle biosynthesis

## Abstract

Abscission zone (AZ) development and the progression of abscission (detachment of plant organs) have been roughly separated into four stages: first, AZ differentiation; second, competence to respond to abscission signals; third, activation of abscission; and fourth, formation of a protective layer and post-abscission trans-differentiation. Stage three, activation of abscission, is when changes in the cell wall and extracellular matrix occur to support successful organ separation. Most abscission research has focused on gene expression for enzymes that disassemble the cell wall within the AZ and changes in phytohormones and other signaling events that regulate their expression. Here, transcriptome data for soybean, tomato and Arabidopsis were examined and compared with a focus not only on genes associated with disassembly of the cell wall but also on gene expression linked to the biosynthesis of a new extracellular matrix. AZ-specific up-regulation of genes associated with cell wall disassembly including cellulases (beta-1,4-endoglucanases, *CELs*), polygalacturonases (*PGs*), and expansins (*EXPs*) were much as expected; however, curiously, changes in expression of xyloglucan endotransglucosylase/hydrolases (*XTHs*) were not AZ-specific in soybean. Unexpectedly, we identified an early increase in the expression of genes underlying the synthesis of a waxy-like cuticle. Based on the expression data, we propose that the early up-regulation of an abundance of small pathogenesis-related (*PR*) genes is more closely linked to structural changes in the extracellular matrix of separating cells than an enzymatic role in pathogen resistance. Furthermore, these observations led us to propose that, in addition to cell wall loosening enzymes, abscission requires (or is enhanced by) biosynthesis and secretion of small proteins (15–25 kDa) and waxes that form an extensible extracellular matrix and boundary layer on the surface of separating cells. The synthesis of the boundary layer precedes what is typically associated with the post-abscission synthesis of a protective scar over the fracture plane. This modification in the abscission model is discussed in regard to how it influences our interpretation of the role of multiple abscission signals.

## Introduction

The process of abscission is roughly divided into four sequential stages with slight modifications by different authors (Patterson, 2001; Estornell et al., 2013): first, abscission zone (AZ) differentiation; second, competence to respond to abscission signals; third, activation of abscission; and fourth, formation of a protective layer and post-abscission trans-differentiation. It was recognized many years ago, based on light and electron microscopy, that during the activation of abscission (i.e., abscission stage 3) AZ cells expand at the fracture plane and the cells separate along the middle lamella (Hall and Sexton, 1974). It has been assumed that the swelling of these cells helps to create the forces necessary to break vascular connections (i.e., rigid xylem vessels), which then allows the organs to separate. Many genes and proteins linked to cell wall loosening and degradation of the middle lamella including cellulases (beta-1,4-endoglucanases, CELs), polygalacturonases (PGs), xyloglucan endotransglucosylase/hydrolases (XTHs), and expansins (EXPs) have been identified and characterized from AZ of several different plant species (Roberts et al., 2002). Also commonly observed at this stage in organ abscission is an up-regulation of defense genes, which includes pathogenesis-related (*PR*) genes (Del Campillo and Lewis, 1992; Meir et al., 2011; Gonzalez-Carranza et al., 2012; Estornell et al., 2013). The role of *PR* gene expression during abscission is often assumed to protect vulnerable abscising cells from opportunistic pathogen invasion (Del Campillo and Lewis, 1992).

In addition to cell wall loosening and *PR* genes, a great deal of attention has been directed toward the identification and characterization of signals that initiate abscission (Taylor and Whitelaw, 2001; Roberts et al., 2002; Liljegren, 2012). Phytohormones and other signals that have been shown to modulate abscission are many-fold: ethylene, auxin, abscisic acid, jasmonic acid, and IDA (a small secreted peptide named INFLORESCENCE DEFICIENT IN ABSCISSION) (Taylor and Whitelaw, 2001; Roberts et al., 2002; Liljegren, 2012; Kim et al., 2013). These abscission signals, however, may not all act in synchrony but sequentially or independently (Patterson and Bleecker, 2004). It is not our intention here to evaluate the role of a multitude of abscission signals, but rather focus on the cellular processes that these signals initiate and regulate. A more complete understanding of the processes and mechanisms utilized for successful organ separation will help to clarify the interactions and interdependencies of the various signals that regulate these processes.

Technological advances have made it possible to now obtain expression results for the entire transcriptome of any desired tissue or developmental program (Zhong et al., 2011; Grassi et al., 2013). In the present work we performed RNA-seq of soybean leaf AZs (LAZ) and the petioles after the AZs were removed (non-AZ petiole, NAZ-pet) harvested from explants exposed to ethylene for 0, 12, 24, 48, and 72 h, and compared the results to microarray data for RNA collected from tomato flower pedicel AZ (FAZ) and proximal non-AZ pedicel (NAZ) at 0, 4, 8, 12, 16, and 20 h after removal of the flowers. In addition, we compared the soybean and tomato results to RNA-seq results for the transcriptomes of Arabidopsis flower receptacles from wild type (WT) and the *hae-hsl2* double mutant (Niederhuth et al., 2013). If left untouched, the petals, sepals and stamens of the *hae-hsl2* mutant do not abscise (Niederhuth et al., 2013) The Arabidopsis data is not a time-course study of differential gene expression during floral organ abscission; nevertheless, the AZ tissues (receptacles) were collected at an early stage of abscission (stage 15) when changes in gene expression for cell separation were first evident in the WT AZ, and, therefore, useful for comparing to early stages of soybean and tomato abscission. Inclusion of the Arabidopsis results in comparison to the soybean and tomato results further highlights shared processes associated with abscission and also the independence and interdependence of regulatory pathways controlling abscission.

We focus here on genes associated with the disassembly and modification of the primary cell wall of the AZ cells, and also the synthesis of a new and different extracellular matrix on the AZ cells as abscission progressed. As expected, we found many homologous and orthologous genes annotated as cell wall loosening and PR proteins that were previously reported to be up-regulated during abscission in many species (Tucker et al., 1988; Kalaitzis et al., 1997; Roberts et al., 2002; Lashbrook and Cai, 2008; Meir et al., 2010). Of special interest here was the early and abundant expression in all three transcriptomes of genes encoding small (15–25 kDa) secreted proteins and genes associated with the deposition of a wax-like cuticle. The expression profiles for many of these genes were very similar to those for cell wall disassembly, i.e., cellulase, PGs, and EXPs. These observations led us to propose that secretion of small proteins and a wax-like cuticle might play an important role in restructuring the extracellular matrix to facilitate organ separation independent of the deposition of a protective scar after separation is complete. The need to synthesize a boundary layer during separation is discussed in regard to abscission signals and their regulation of gene expression required for successful organ separation.

## Methods

### Plant material

Soybean (*Glycine max*, cv. Williams82) plants were grown in the greenhouse and harvested when the primary leaves were fully expanded (19–24 days). Stem-petiole explants were prepared by cutting the stem approximately 4 cm below the leaf node and removing all but approximately a 5-mm triangular portion of the primary leaf blade (Supplemental Figure S1). Explants were placed in Erlenmeyer flasks with water and put into a darkened chamber wherein 25 μL/L ethylene in air saturated with water was passed through at a rate of 2 L/min at 25°C. In soybean, there is an AZ at the base of the petiole at the juncture with the stem (lower AZ) and another AZ at the distal end of the petiole approximately 1 mm below the leaf blade (upper AZ) (Supplemental Figure S1). In our system with ethylene treatment, the petiole separated from the stem at the lower AZ at approximately 48 h from just the weight of the petiole, but the distal portion of the upper AZ at the top of the petiole, which is rather small and light, did not sometimes fall away from the petiole even after 72 h of ethylene. To assess the extent of separation at the upper AZ, the distal part of the AZ was gently touched with forceps and, if it fell away from the petiole, the AZ was recorded as having fully abscised. Nonetheless, with high humidity, the petiole and AZ continue to senesce after separation from the parent plant, and did not dry out. Approximately 2 mm, which included approximately 1 mm of proximal and distal sides of the upper, leaf AZ (LAZ), was collected from 20 explants (2 upper AZs per explant or 40 AZs total) and flash frozen in liquid nitrogen. After excising the AZ, the petiole ends were again trimmed to avoid incidental collection of any AZ tissue and the petioles (NAZ-pet) were flash frozen. The lower AZ was not collected, because at this stage of growth the petioles are relatively small and we wanted to avoid incidental collection of a portion of the lateral bud that is very close to the lower AZ. Collection of a small part of a lateral bud with a meristem in it would compromise the interpretation of AZ-specific gene expression. AZs and petioles (NAZ-pet) were collected at 0 h (immediately prior to ethylene treatment) and at 12, 24, 48, and 72 h after the ethylene treatment had begun. Plants were grown, explants prepared and samples collected three separate times to give three independent experimental replicates.

Flower clusters (inflorescences) from tomato (*Solanum lycopersicum*) cv. New Yorker were harvested from 4-month-old greenhouse-grown plants between 08:00 and 10:00 a.m. Explants were prepared as previously described (Meir et al., 2010). Briefly, inflorescences bearing at least 2–4 freshly open flowers were brought to the laboratory under high humidity conditions. Closed young flower buds and senesced flowers including the entire pedicel and AZ joint were removed so that only freshly opened flowers and pedicels remained on the inflorescence. The stem ends were trimmed, and groups of 2–3 inflorescence explants were placed in vials containing double distilled water (DDW). Abscission was induced by cutting the flowers off the inflorescence at the base of the receptacle. Cutting off the flowers removes a source of auxin that inhibits abscission (Meir et al., 2010). Tissue samples for RNA extraction were taken from the flower AZ (FAZ) and proximal pedicel (NAZ) of 30 segments. The 2 mm FAZ collection included approximately 1 mm on either side of the AZ joint or fracture once it appeared. Samples were collected at 0 h (immediately before flower removal) and 4, 8, 12, 16, and 20 h after flower removal. Explants were harvested and tissue samples collected twice for two independent experimental replicates.

### RNA sequencing and microarrays

RNA was isolated from soybean LAZ and NAZ-pet after 0, 12, 24, 48, and 72 h of exposure to ethylene using a Qiagen RNeasy Mini Kit following the standard protocol (Qiagen, Germantown, MD, USA). Each experiment produced 10 RNA samples, which resulted in 30 RNA samples for the 3 replicate experiments. Further RNA purification, cDNA synthesis and sequencing on an Illumina GAII sequencer were performed at Cornell University, Ithaca, NY, USA as previously described (Zhong et al., 2011; Grassi et al., 2013). The 30 RNA samples were processed, barcoded and run together on the GAII sequencer. The raw sequence files have been submitted to the NCBI SRA databases with the study accession SRP050050. On average, each RNA sample produced approximately 4 million reads (Supplemental File S2). Raw sequences were trimmed to remove ambiguous ends. Using Bowtie (Langmead et al., 2009), approximately 40,000 (1%) of the reads mapped to ribosomal RNA (rRNA) and were removed from the data set. Using TopHat (Trapnell et al., 2009), approximately 90% of the remaining RNA mapped to a predicted soybean transcriptome (cds) (*G. max* 189 genome assembly). Multiple versions (splice variants) were not taken into account. A single version (usually the last version) was used for alignment. A total of 54,175 transcripts were used for the alignment. The number of reads that aligned to a gene was normalized as Reads Per Kilobase per Million mapped reads (RPKM) (Mortazavi et al., 2008).

For a gene to be counted as expressed in the LAZ or NAZ-pet, we required that the mean RPKM for the three replicates be at least 1.0 or greater in at least one of the treatments. Using a cutoff of 1.0 RPKM resulted in the selection of 37,572 genes as being expressed in the soybean LAZ or NAZ-pet between 0 and 72 h of exposure to ethylene (Supplemental File S2). Then, to avoid ratios with a zero in the numerator or denominator, any RPKM of less than 0.1 was given the minimal value of 0.1. QPCR was performed as previously described (Tucker et al., 2007) on a few selected genes to confirm that the RNA-seq and RPKM normalization produced the expected expression profile (results not show).

The Meir lab at the Volcani Center, Bet-Dagan, Israel has prepared a tomato AZ-specific microarray chip in collaboration with Genotypic Technology Pvt. Ltd., Bangalore, India. Each chip includes 111,718 probe sets for more than 40,000 transcripts. Probe sets were designed using RNA-seq results for pooled RNA samples from non-induced and induced tomato flower pedicel and AZ tissue as described above. However, for the RNA sequencing, the tissues were collected from *Solanum lycopersicum* cv. “VF-36.” Transcriptome libraries for sequencing were constructed according to the Illumina TruSeq RNA library protocol outlined in “TruSeq RNA Sample Preparation Guide” (Part # 15008136; Rev. A, Illumina, USA). The DNA obtained from the prepared libraries was denatured and sequenced on the Illumina Genome Analyzer IIX, using the sequencing by synthesis method to read 72 bases per end. The raw sequencing data were then extracted from the server using the proprietary Illumina pipeline software to obtain a sequence data set in a Fastq format. Quality check of raw data was performed using SeqQC –V2.0 program. The raw sequence data and array information were submitted to the Gene Expression Omnibus (GEO) at the National Center for Biotechnology Information (NCBI) with GEO IDs GSE45355 and GSE45356, and array ID AMADID:043310. Expression was validated by qPCR (results not shown).

For the tomato microarray results, a gene was considered to be expressed, if the minimum mean signal for two replicates was >10 in at least one time point in either the NAZ or FAZ. This selection criterion resulted in the selection of 26,527 genes as being expressed in the FAZ or NAZ. Then, to avoid ratios with a zero in the numerator or denominator, any signal of less than 1.0 was given the minimal value of 1.0.

The RNA-seq results reported here for Arabidopsis WT and the *hae-hsl2* mutant are a reformulation of data from Additional file 2 in Niederhuth et al. (2013). Arabidopsis receptacles, which included the floral organ AZs, were collected as described (Niederhuth et al., 2013). To conform to the analysis of the soybean and tomato transcriptomes, the log2 fold change was reversed to WT over *hae-hsl2*. Similar to the other data sets, a minimum of >10 reads per gene was required to consider the gene as being expressed in either WT or the mutant, and any reads per gene of less than 1.0 was given a minimal value of 1.0 to avoid complications arising from having a zero in the numerator or denominator (Supplemental File S4).

## Results and discussion

### Differences in experimental design

The objective of this study was to identify processes directly linked to successful organ separation that are common to a variety of abscission systems. However, how abscission data is collected is not done the same by all researchers. Therefore, what first needs to be addressed and fully understood are the differences in experimental approaches used to study abscission in soybean, tomato and Arabidopsis. The explant system used to study abscission in soybean and tomato was established many years ago (Addicott, 1982; Sexton and Roberts, 1982). It is a versatile system in which a variety of hormones and chemicals can be exogenously applied. An important part of the explant system is the removal of the source of auxin, i.e., soybean leaves or tomato flowers. Moreover, in soybean and tomato, ethylene is essential for leaf and flower abscission, respectively (Lanahan et al., 1994; Meir et al., 2010; Tucker and Yang, 2012). In the soybean experiments, the explants were continuously exposed to a high concentration of ethylene (25 μL/L), which synchronizes and accelerates abscission, but also induces senescence and ethylene-regulated defense gene expression (Abeles et al., 1992). After a 72 h exposure to ethylene, both the petiole and AZ turned yellow. Thus, treating the entire explant with ethylene removes the variable of none uniform synthesis of ethylene between the AZ and petiole, and reduces differential gene expression linked solely to ethylene.

In the tomato experiments, the inflorescences were not treated with ethylene. However, because ethylene is essential for tomato flower abscission (Lanahan et al., 1994; Meir et al., 2010), it is likely that upon removal of the auxin source ethylene was synthesized in the inflorescence but the synthesis of ethylene may not have been equal in the FAZ and NAZ (discussed later). Nonetheless, the tomato system reflects a more natural process that would occur during flower abscission on the intact plant.

The leaves and flowers of Arabidopsis do not normally abscise and, therefore, Arabidopsis abscission scientists study the abscission of floral organs, petals, stamens, and sepals (Bleecker and Patterson, 1997; Patterson, 2001; Butenko et al., 2003). The Arabidopsis data we used here was collected and published by Niederhuth et al. (2013). They collected flower receptacles from WT and the double mutant *hae-hsl2* at developmental stage 15 flowers. HAE and HSL2 are redundant receptor-like kinases that bind the IDA (INFLORESCENCE DEFICIENT IN ABSCISSION) signaling peptide (Cho et al., 2008; Stenvik et al., 2008; Butenko et al., 2014). In the *ida* mutant, as in the *hae-hsl2* double mutant, the floral organs do not abscise if they are left untouched (Butenko et al., 2003). At flower stage 15 both WT and mutant flowers are fully open but the petals have not abscised (Niederhuth et al., 2013). Stage 15 flowers correspond to approximately the same developmental stage as flowers at positions 3 and 4 used in other studies of Arabidopsis floral organ abscission (Patterson and Bleecker, 2004; Cai and Lashbrook, 2008; Cho et al., 2008; Basu et al., 2013; Liu et al., 2013). In wild-type flowers at positions 3–4 abscission-associated gene expression has already begun and the break-strength of the petals has started to decline but the petals have not fallen off under their own weight (Kim and Patterson, 2006; Cai and Lashbrook, 2008; Niederhuth et al., 2013). The levels of auxin and ethylene in this system are unknown. Nonetheless, ethylene can accelerate abscission of Arabidopsis floral organs (Gonzalez-Carranza et al., 2007) but it does not appear to be essential because floral organ abscission is only delayed in ethylene-insensitive mutants (Patterson and Bleecker, 2004). Moreover, auxin also plays a role in Arabidopsis floral organ abscission (Basu et al., 2013). If auxin levels are genetically up-regulated or down-regulated in the AZ of Arabidopsis floral organs, abscission is delayed or accelerated, respectively (Basu et al., 2013). Thus, although the experimental approach to study Arabidopsis abscission is different from soybean and tomato and ethylene appears not to be essential to Arabidopsis floral organ abscission, comparison of differential gene expression in the transcriptomes of WT and the *hae-hsl2* mutant to soybean and tomato provides additional information and confirmation of conserved molecular and metabolic processes that contribute to organ separation.

### Overview of differential gene expression

**Figure 1:**
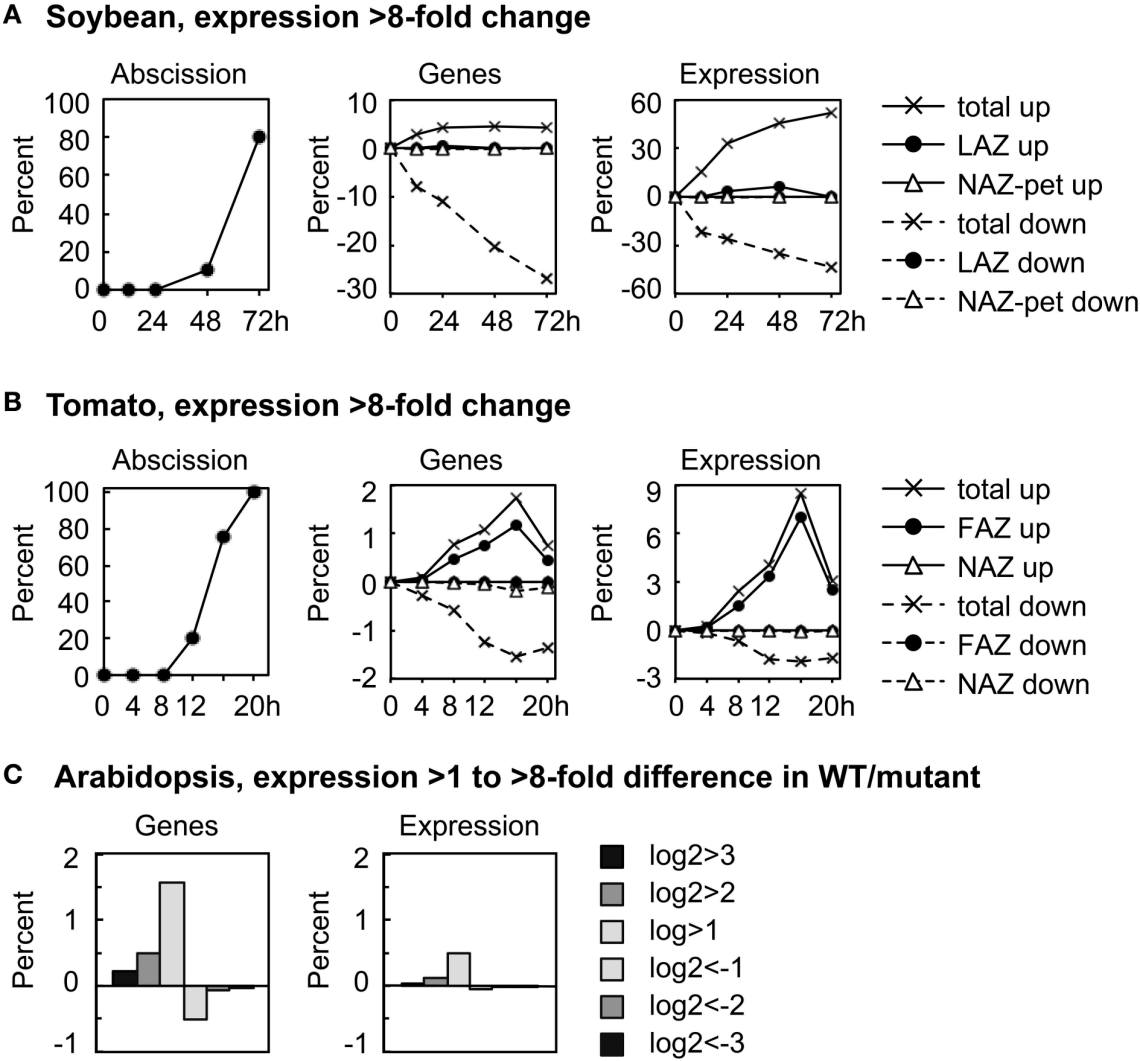
**Overall perspective on major changes in gene expression during abscission of soybean leaves, tomato flowers and Arabidopsis floral organs**. **(A)** The percent of soybean pulvinar leaf abscission and the percent of genes with greater than 8-fold change (log2 >3 or < −3) in expression relative to the total number of genes expressed (37,572) and the differential expression of the same genes relative to the average total RPKM for each RNA sample (666,213 RPKM). **(B)** Similar to **(A)** except that tomato flower abscission had 26,572 expressed genes with a total average microarray signal of 64,696,620. **(C)** The percent of expressed genes in wild-type Arabidopsis receptacles relative to genes in the double mutant *hae-hsl2* and the differential expression of the same genes (Niederhuth et al., 2013). In the Arabidopsis data, 20,883 genes were counted as expressed with a total mean of 38,770,114 reads. Because differential expression was much less in the Arabidopsis data, 2-, 4-, and 8-fold differences are plotted as a bar graph.

We plotted the overall change in expression for those genes that changed markedly over time in the AZ and NAZ of soybean and tomato and between WT and *hae-hsl2* of Arabidopsis. For soybean and tomato we used an arbitrary threshold for a change in gene expression of >8-fold (log2>3 or <-3) (Figure 1). In soybean where the auxin source has been removed and the explants treated with ethylene, approximately 5% of all the expressed genes displayed an increase in expression of >8-fold in both the AZ and petiole; however, the expression of a much greater number of genes, 30%, decreased >8-fold (Figure 1A). Although 5% of all the genes increased and 30% decreased, only about 1% of the genes were expressed in an AZ-specific manner. Interestingly, at 72 h the 5% of the genes that increased in expression accounted for 50% of the transcripts in the transcriptome of both the AZ and petiole (Figure 1A). There was also a significant increase in transcripts that were AZ-specific but still much less than the total. In tomato, there was a similar but much smaller decline in gene expression that is common to both AZ and NAZ (Figure 1B). In tomato flowers, which abscise much more rapidly than the soybean leaves, approximately 2% of the genes changed expression >8-fold by 16 h, which accounted for almost 9% of the transcripts in the transcriptome; however, 90% of the gene expression was AZ-specific (Figure 1B). A possible explanation for why most of the differential expression >8-fold was AZ-specific in tomato may be that ethylene synthesis was greater in the AZ than the NAZ (discussed later).

In Arabidopsis, the magnitude of differential gene expression between WT and the mutant was relatively small. Less than 2% of the genes increased in expression more than 2-fold, log2>1, which accounted for only a 0.5% change in the total transcriptome (Figure 1C). There are at least two explanations for the small difference in gene expression between the WT and *hae-hsl2* receptacles. First, the receptacle collection was an early stage of abscission, and, second, the IDA signaling path is one of several signals that regulate gene expression in Arabidopsis abscission (discussed later).

**Figure 2:**
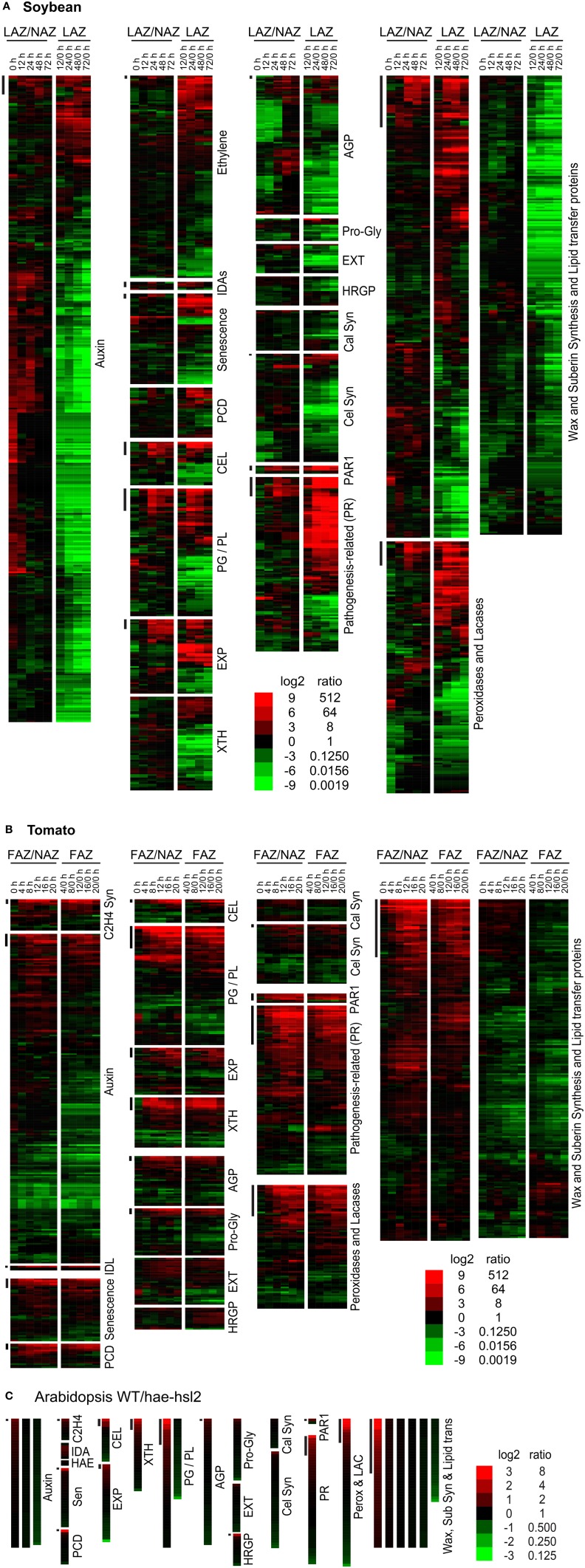
**Heat map display of expression profiles for genes in soybean leaf pulvinus AZs (LAZ) and petioles with the AZs removed (NAZ-pet), tomato flower AZs (FAZ) and proximal pedicel (NAZ) and Arabidopsis wild type (WT) and ***hae-hsl2*** mutant receptacle AZs**. **(A)** Soybean, **(B)** tomato, and **(C)** Arabidopsis gene expression. The expression profiles are presented as ratios for AZ-specificity (AZ/NAZ), and as ratios for AZ expression at different time intervals relative to 0 h (start of ethylene treatment of soybean or removal of auxin source for tomato), e.g., 12/0, 24/0, 48/0, or 72/0 h. The bars at the top left of each category mark genes whose expression is >8-fold AZ-specific (AZ/NAZ) and >8-fold up-regulated (time interval/0 h). The scale at the bottom indicates the color representing the log2 ratio and ratio listed next to it. The color scale for Arabidopsis is different than soybean and tomato because differential expression was less in the Arabidopsis tissues. The genes in each category, their nomenclature, log2 ratios, and annotations are given in Supplemental Files S2–S4 for soybean, tomato and Arabidopsis, respectively.

**Figure 3:**
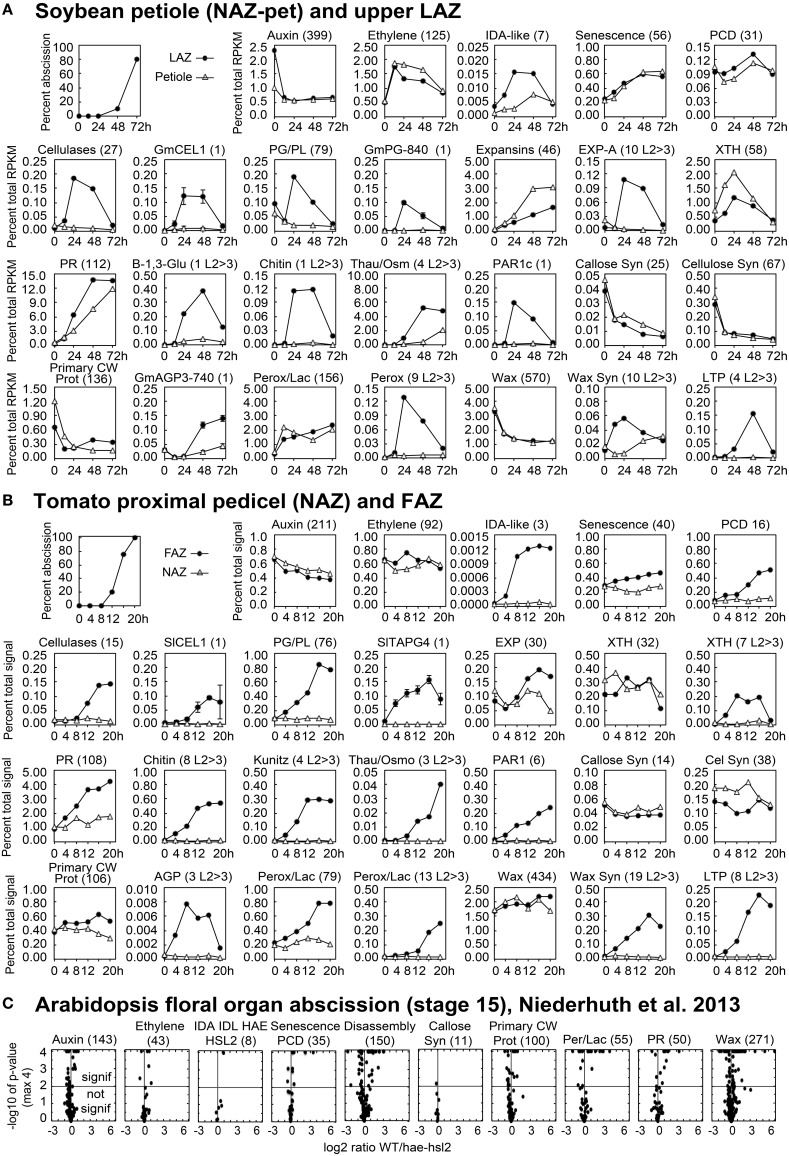
**Line graphs (soybean and tomato) and volcano plots (Arabidopsis) showing the change in gene expression for selected groupings of genes and some individual genes**. The percent of abscission is indicated in the graph at the top left corner the sections for **(A)** soybean and **(B)** tomato as a reference. **(A)** The Y-axes indicate the percent expression relative to total RPKM for all 37,572 expressed soybean genes (transcriptome). The X-axes indicate the length of exposure to ethylene. Solid circles represent expression in the LAZ and the open triangles in petioles (NAZ-pet). **(B)** Similar to **(A)** but The Y-axes indicate the percent expression relative to total microarray signal for 26,527 expressed tomato genes. The X-axes indicate the hours after removal of the flowers, auxin source. Solid circles represent expression in the FAZ and the open triangles in NAZ. The number within the parenthesis to the right of each category or gene name is the number of genes included for that particular graph. Plots with an L2>3 after the number in parentheses show the cumulative expression for only those genes with a significant 8-fold increase in expression (*p* < 0.015 and log2>3 for any time relative to 0 h). Standard error bars cannot be calculated for plots of multiple genes. **(C)** Volcano plots of differential expression in WT/*hae-hsl2* receptacles at developmental stage 15. Y-axes are the −log10 of the *p*-values. A *p* < 1% (<0.015) is a *p*-value of −log10 >2, which is indicated by the horizontal line through the middle. Any expression with a *p*-value (significance) of less than 0.0001 (−log10>4) was set to a minimum of 0.0001, which is why many points align across the top. The X-axes are the log2 ratios for expression (reads) of WT/*hae-hsl2*. The genes in each category, their nomenclature, log2 ratios, and annotations are given in Supplemental Files S2–S4 for soybean, tomato, and Arabidopsis, respectively.

The leaves and flowers were removed from the soybean and tomato explants, respectively, and, as expected, the removal of the auxin source is reflected in a general decline in auxin associated gene expression in both the AZ and NAZ (Figures 2, 3). Nonetheless, there were a few auxin-associated genes that increased in an AZ-specific manner in both soybean and tomato (Figures 2, 3) and many of these were linked to auxin movement (e.g., PIN) or auxin conjugation (e.g., GH3) (Supplemental Files S2, S3).

The soybean explants were treated with ethylene and because of this there was an expected non AZ-specific increase in gene expression for ethylene-associated genes linked to senescence and programmed cell death (PCD) (Figures 2A, 3A). There was also an expected increase in ethylene-induced defense gene expression (*PR* gene expression) (Figures 2A, 3A); nonetheless, several *PR* genes were expressed AZ-specifically (see below). In tomato, the increase in ethylene-associated genes (senescence, PCD and *PR* genes) was mostly AZ-specific (Figures 2B, 3B). In both soybean and tomato there was an AZ-specific increase in expression of genes for ethylene synthesis (i.e., *ACS* and *ACO*) (Supplemental Files S2, S3). This would suggest that in tomato ethylene synthesis was AZ specific whereas in soybean, because the explants were treated with a high concentration of ethylene, the AZ-specific synthesis of ethylene was inconsequential. Thus, in tomato, ethylene-induced gene expression was AZ-specific due to the synthesis of ethylene in the AZ tissue.

### Changes in the transcriptome linked to cell wall disassembly

Genes within the categories for cellulases (*CELs)*, pectinases (*PGs*) and pectin lyase-like (*PGs* and *PLs*), *EXPs, XTHs* were selected based primarily on their annotation, which is most often derived from the most similar gene sequence in Arabidopsis. The category labeled cellulases included any gene annotated as cellulase or glycoside hydrolase (GH) family 9, but genes in the subfamily GH9A1 were placed into the cellulose biosynthesis category, which is more appropriate for *KORRIGAN-like* cellulase genes (Doblin et al., 2002).

As expected, AZ-specific gene expression for cell wall disassembly proteins increased markedly in soybean, tomato and Arabidopsis (Figures 1, 2). Of interest in regard to cell wall disassembly was an unexpected result for *XTH* expression in soybean leaf abscission. Transmission electron micrographs indicate that cellulose microfibrils (striations) are untethered during abscission but not degraded (Hall and Sexton, 1974). It is generally accepted that a xyloglucan network plays an important role in tethering cellulose microfibrils and, along with pectins distributed within the primary cell wall, may influence the free movement of proteins and other large compounds across the primary cell wall out to the middle lamella (Carpita and Gibeaut, 1993; Cosgrove, 2001). It was surprising to find that in soybean no *XTHs* were up-regulated in an AZ-specific manner and most *XTHs* declined (Figure 2). However, in tomato and Arabidopsis there was an AZ-specific increase in expression of *XTH*. Ethylene treatment of soybean may be part of the reason for this difference. It is plausible that XTHs also play a role in in ethylene-induced senescence and this is why XTH was not LAZ-specific. However, there is another possibility. Cellulase (beta-1,4-endoglucanase) activity is assayed using carboxymethyl cellulose, which is a soluble cellulose derivative (Urbanowicz et al., 2007). *In vitro* assays with purified plant cellulases by themselves do not degrade crystalline cellulose microfibrils. The *in vivo* substrates for cellulases are not known. Therefore, it is possible that some cellulases may cleave chains of beta-1,4-glucans within xyloglucan polymers that fix the cellulose microfibrils in place (Hayashi and Kaida, 2011; Eklöf et al., 2012), or they cleave beta-1,4-glucans at the surface of the microfibrils that play a role in tethering and crosslinking the microfibril network. Transcription of *GmCEL01* increased earlier than other cell wall loosening enzymes during soybean abscission (Figure 3A). It is possible that the role of the GmCEL1 enzyme is to loosen the hemicellulosic fraction (xyloglucans, etc.) or their tethering to the cellulose microfibrils, which then opens up the cell wall for movement of protein and other compounds out to the middle lamella.

A heat map (Figure 2) is useful to display a general perspective on the expression patterns within each category of genes, but it does not take into account transcript abundance. For example, *GmCel01*, (Glyma11g02350, accession U34755) accounted for approximately 75% of the RNA for the 27 *CELs* expressed in the soybean LAZ (Figure 3A). The same was true for tomato where *SlCEL1* accounted for 75% of the transcript for 17 *CELs* (Figure 3B). Similarly, one or just a few *PGs* accounted for most of the transcript in both soybean and tomato AZ (Figures 3A,B). This was not necessarily the case for other gene families (Figure 3 and Supplemental Files S2–S4).

Expression of EXPs in abscission was more complex. In soybean, *EXPs* increased markedly in both the LAZ and petiole but only alpha *EXPs* were AZ-specific, but the AZ-specific expression of alpha *EXPs* was less than 10% of the overall expression for *EXPs* (Figure 3A). *EXP* expression in tomato had an AZ-specific component to it but the level of *EXP* gene expression was already relatively high at 0 h (Figure 3B). Based on our results, the role of EXPs in abscission is not easily interpreted.

Gene expression for cell wall disassembly in Arabidopsis abscission will be discussed in greater detail later. Nonetheless, the expression of *CELs, PGs, EXPs*, and *XTHs* were significantly higher in WT than the mutant (Figure 3C and Supplemental File S4). What was most surprising in contrast to soybean and tomato abscission was that the level of gene expression for all of the cell wall disassembly genes in both the WT and the *hae-hsl2* mutant started at fairly high levels (Supplemental File S4).

### Expression of cellulose synthases and typical primary cell wall proteins

We considered the possibility that the cell wall and middle lamella of fracture plane cells would be degraded but once separation was complete the same cells would synthesize a new cell wall much like the previous one that included new cellulose microfibrils, hemicelluloses and protein. The primary wall of a plant cell consists of approximately 20% protein (Carpita and Gibeaut, 1993). To assess whether or not a typical cell wall was synthesized we examined the expression of cellulose synthesis genes and typical primary cell wall proteins, extensins (EXTs), arabinogalactan proteins (AGPs), proline and glycine-rich proteins, and hydroxyproline-rich glycoproteins (HRGPs). In soybean, the expression of almost all of these genes declined (Figures 2A, 3A); however, in tomato and Arabidopsis, this grouping of genes were mostly unchanged or increased slightly (Figures 2B,C, 3B,C). Based on these observations, we conclude that cellulose synthesis and synthesis of typical primary cell wall proteins are not a significant part of abscission or formation of a protective layer.

### Synthesis of a more proteinaceous extracellular matrix

Overall there was a strong up-regulation of *PR* genes. It might seem odd to include *PR* gene expression under the sub-heading of synthesis of a new and different extracellular matrix because the role of *PR* gene expression during abscission is customarily assumed to protect the vulnerable abscising cells from opportunistic pathogen invasion (Del Campillo and Lewis, 1992). However, the first and possibly the most important defense against pathogens is a structural barrier, which includes the cell wall and cuticle (Hamann, 2012). However, at the site of a microbial infection, the cell wall is further reinforced by the synthesis of papillae (Voigt, 2014). Callose, a 1-3 linked beta glucan polymer, is major component of papillae. Because gene expression for callose synthesis is either down-regulated or unchanged in all three abscission systems, we conclude that callose is not a primary component in the new extracellular matrix (Figures 2, 3). Nonetheless, what is important to successful organ separation is the creation of a flexible barrier to pathogens that both inhibits infection and allows cell expansion. We argue that in addition to an enzymatic role in the defense against pathogens, a remodeled extracellular matrix containing an abundance of small PR proteins is important to the actual separation process. An extracellular matrix made up of cross-linked protein rather than long chains of polysaccharides, i.e., callose, cellulose or hemicelluloses, might better allow for cell enlargement that creates the physical stress across the fracture plane, but still provide enough structure to prevent the fracture plane cells from rupturing.

What is the PR protein composition of the extracellular matrix? It does not appear to be the same in each of the three systems. In soybean, *thaumatin* begins to increase early and by 48 h expression of *thaumatin* accounted for 5% of the AZ transcriptome (Figure 3A and Supplemental File S2). Expression of *Chitinase* and *beta-1,3-glucanase* is also greatly up-regulated in an AZ-specific manner. In tomato, *chitinase* and *kunitz trypsin inhibitor* are abundantly expressed in the AZ but *thaumatin* is not as strongly expressed (Figure 3B). In Arabidopsis, *thaumatin, kunitz trypsin inhibitor*, and *chitinase* are all more highly expressed in WT than the mutant (Supplemental File S4).

*PAR1* (*photoassimilate-responsive-1*) is also considered to be a *PR* gene (Herbers et al., 1995). We have separated *PAR1s* from the others because they exemplify our view that synthesis of a new protein-rich extracellular matrix is important to the separation process. *PAR1s* are expressed in an AZ-specific manner in all three systems with an expression profile much like the AZ-specific *CELs* and *PGs*. PAR1s are small proteins (approximately 18 kDa) with no known enzymatic function but have several cysteines that might support protein cross-linking in the cell wall. Also, *PAR1s* are highly conserved in both dicots and monocots (Supplemental File S5).

### Synthesis of a waxy cuticle

Now, to detail and discuss our observation that was somewhat unexpected for us because it is not commonly discussed in the abscission literature as being a part of the separation process but rather a part of the synthesis of a protective layer after separation has occurred. We found an early AZ-specific increase in gene expression of several genes that are best linked to the synthesis and secretion of a waxy cuticle. Because both wax and suberin biosynthesis require protein for lipid modification and transport and phenylpropanoid metabolism, we have also included suberin-associated genes in this category. Genes included in the wax-suberin category were selected based on the cuticle synthesis genes listed by Suh et al. (2005), suberin biosynthesis genes listed by Soler et al. (2007) and genes whose gene ontology (GO) annotation included terms for lipid or fatty acid synthesis or modification. Peroxidases are also a part of wax and suberin synthesis. Gene expression for several peroxidases and laccases increased markedly during abscission and some quite early. Although peroxidases and laccases may be involved in the synthesis of waxes and suberin, they may also be tied to cross-linking of protein and pathogen defense (Matheis and Whitaker, 1984; Almagro et al., 2009). For this reason, we have put *peroxidase* and *laccase* genes into their own category. Nonetheless, collectively, we conclude that independent of the category they are included in peroxidases and laccases are a part of creating a new and different extracellular matrix.

In soybean, there was more than a 50% decline in the expression of the 570 genes in the wax-suberin category (Figure 2A). This is probably because cuticle synthesis is not generally required for senescing tissue. However, there was a subset of 10 wax synthesis genes that were significantly up-regulated in the AZ more than 8-fold within the first 12 h (Figure 2A). This was significantly earlier than the increase for *CELs* or *PGs*. Three of the 10 wax synthesis genes encoded GDSL-like lipases, which are important to cuticle synthesis (Yeats and Rose, 2013), and the most AZ-specific of the 10 was a *CER4*, Jojoba acyl CoA reductase, which is also important to the synthesis of a wax cuticle (Rowland et al., 2006) (Supplemental File S2). In addition to fatty acid and wax synthesis, there was a strong AZ-specific increase in four lipid transfer proteins (LTP) (Figure 3A). In tomato, we found 19 wax synthesis and 8 *LTP* genes that increased specifically in the AZ more than 8-fold (Figure 3B). Like soybean, the most AZ-specific wax synthesis gene in tomato was a *CER4*, Jojoba acyl CoA reductase (Supplemental File S3).

Gene expression for wax synthesis and lipid transfer is particularly interesting in the Arabidopsis data. This category of genes (wax-suberin) had the greatest difference between the WT and the *hae-hsl2* mutant (Figure 3C and Supplemental File S4). Moreover, if one sorts the original published Additional file 2 (fold-change calculated for the opposite ratio, i.e., mutant/WT) the gene showing the greatest difference between WT and the mutant was a *bifunctional inhibitor/lipid-transfer protein* (AT4G22485.1) and the third gene in the sorted file was a *GDSL-like lipase* (AT5G03810.1) (Niederhuth et al., 2013). Both of these cuticle-associated gene families are strongly up-regulated and AZ-specific in soybean and tomato (Supplemental Files S2, S3). When the original Arabidopsis Additional file 1 was sorted this way, the first cell wall disassembly gene, *PGAZAT* (AT2G41850), was not even in the top 50 differentially expressed genes. The large differential expression of putative cuticle synthesis genes in the Arabidopsis data may be significant to an interpretation of the role of IDA signaling in abscission (discussed below).

It should be noted that expression of *LTPs* in abscission has been previously reported (Agusti et al., 2009; Nakano et al., 2013). Here we put *LTPs* expression in context with additional observations and propose that synthesis of a waxy-like cuticle is important to successful organ separation.

### Comparison to separation during organogenesis

Based on our abscission transcriptome results, it appears that an early up-regulation of gene expression for synthesis and secretion of a waxy cuticle-like substance during abscission is a common feature for abscission in many species. One possible explanation for this is that the separating cells need to be protected from water loss (Samuels et al., 2008). Also, synthesis of a cuticle could be part of a defense mechanism (Samuels et al., 2008). However, there is another possible role for synthesis of a waxy cuticle. Arabidopsis has proven to be an excellent model system for the study of cuticle synthesis (Samuels et al., 2008; Shi et al., 2011; Yeats and Rose, 2013). Interestingly, a common phenotype for knockout mutants for transcription factors and other genes related to the synthesis of the cuticle is that organs remain fused or display structural anomalies (Shi et al., 2011; Yeats and Rose, 2013). Fused floral organs were also observed in a wax-deficient mutant in tomato (Smirnova et al., 2013). The composition of the extracellular matrix around boundary cells that separate organs in the meristem is not well understood (Žádníková and Simon, 2014). Nevertheless, based on the genetic results, it seems likely that the synthesis of a cuticle like substance plays a role in the separation of organs in the meristem. It seems plausible that a similar process might be implemented during abscission of leaves, flowers, fruit, or floral organs. Moreover, expression of meristem-associated developmental genes in AZ seems to be consistent with this hypothesis (Wang et al., 2013). It should also be noted that modification of pectin structure was found to play a role in organogenesis (Peaucelle et al., 2011). Organogenesis appears to have many features in common with abscission.

### Synthesis of a boundary layer and hormonal and IDA-like signaling

If the synthesis of a waxy, cuticle boundary is important to successful organ separation, you might assume someone would have identified Arabidopsis mutants that were linked to this function. We suggest that the *ida, hae-hsl2* mutants are more closely linked to the synthesis of a cuticle-like boundary layer than expression of genes for cell wall disassembly. To explain, first, although we do not have a time course comparison of WT and *hae-hsl2*, differential expression of cuticle synthesis and peroxidases genes was greater in this data than for cell wall disassembly genes (Figure 3C and Supplemental File S4) (Niederhuth et al., 2013). Moreover, break-strength measurements for the *ida* and *hae-hsl2* mutants indicate that IDA-signaling affects only a part of the separation process (Cho et al., 2008; Liu et al., 2013). Break-strength is a measure of the force needed to pull the distal organ, e.g., petal, away from the proximal organ, e.g., receptacle (Craker and Abeles, 1969; Del Campillo and Bennett, 1996). In WT flowers, the petal break-strength begins to decline at flower position 3 soon after the flowers open (Patterson and Bleecker, 2004). Break-strength of WT petals continues to decline until positions 6–8 when the petals, stamens and sepals fall off from their own weight. In the *ida* mutant (Butenko et al., 2003), which has an abscission phenotype very similar to the *hae-hsl2* mutant (Cho et al., 2008), the petal break-strength also begins to decline at position 3 but declines at a slightly slower rate than WT; however, between flower positions 8–12 the petals begin to re-adhere to the receptacle so that by position 22 the petals are senescent but have regained a break-strength approximately equal to that of a flower at position 2 (Butenko et al., 2003). Scanning electron micrographs (SEM) of the fracture plane cells of WT petals on flowers at positions 10 show nicely rounded and enlarged cells. SEM micrographs of the petal fracture plane of *ida* at flower position 10, when break-strength was at its lowest, displayed partially enlarged and rounded cells (Butenko et al., 2003). However, at flower position 22 of the *ida* mutant, after the petals had re-adhered to the receptacle and were forcibly pulled off, the micrographs of the fracture plane displayed cells that were broken and torn (Butenko et al., 2003). Clearly, part of the abscission process is implemented in *ida* but the process is incomplete. The *nevershed* (*nev*) mutant, that has a defect in the cellular secretion process through the golgi apparatus, also displays a V-shaped break-strength profile; however, the cells in the fracture plane at the lowest break-strength appear to enlarge even more than in the WT cells (Liu et al., 2013). The phenotypes of *ida, hae-hsl2*, and *nev* are similar but crosses between them suggest that *NEVERSHED* expression is not solely dependent on IDA signaling (Liu et al., 2013). Nevertheless, the *nev* mutant may also affect secretion of components needed for synthesis of a waxy cuticle more than cell wall disassembly.

**Figure 4:**
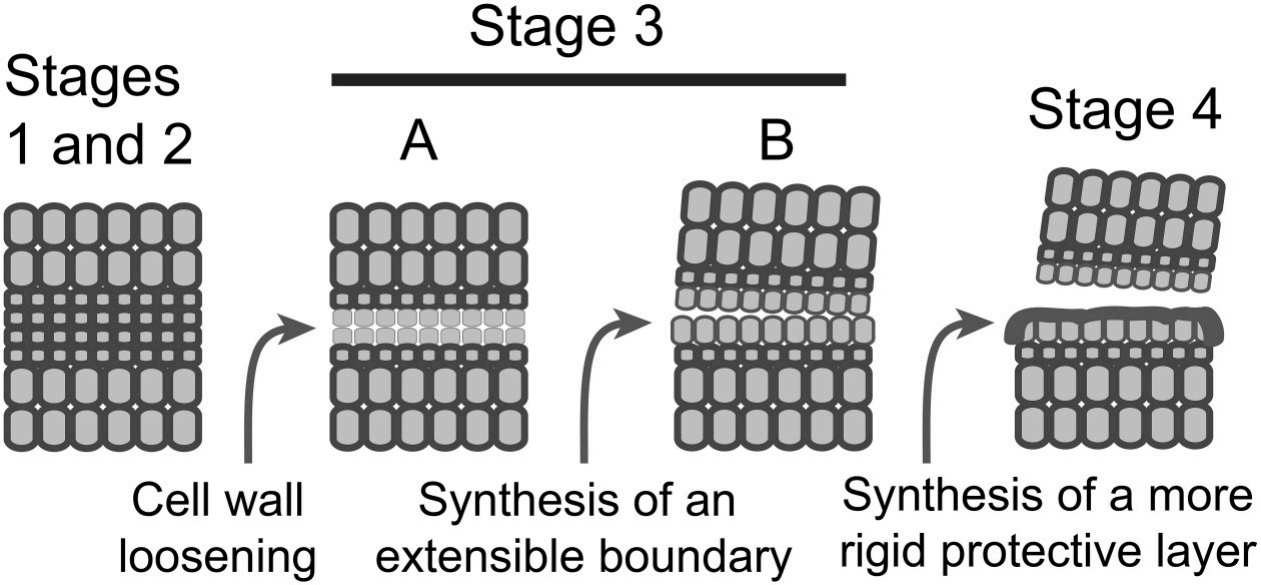
**A schematic model depicting the sequence of events in AZ formation and organ separation**. Stages 1 and 2, AZ differentiation and competence to abscise are combined; Stage 3, activation of abscission, is divided into two temporally overlapping sub-stages, cell wall loosening and synthesis of a boundary layer; Stage 4, synthesis of a protective layer and post-abscission trans-differentiation.

It is possible based on the results and interpretations we have presented here that IDA signaling in Arabidopsis plays a greater role in the synthesis of a new boundary layer than cell wall disassembly and that this boundary layer appears to aid in successful floral organ separation. Can we propose a similar role for IDA in soybean, tomato and other abscission processes? *IDA*-like genes are highly conserved in dicots and also found in some monocots (Vie et al., 2015). In soybean leaf abscission and tomato flower abscission there is an AZ-specific up-regulation of *IDA*-like gene expression (Figures 2, 3 and Supplemental Files S2, S3). However, nobody to the best of our knowledge has demonstrated that IDA signaling is necessary for successful organ separation in any species other than Arabidopsis. It is worth mentioning here that we have used virus-induced gene silencing (VIGS) to suppress *IDA*-like expression in soybean and did not observe any effect on leaf abscission (results not shown). Regulation of abscission is a nexus of signaling events including ethylene, auxin, IDA, and others (Taylor and Whitelaw, 2001; Gonzalez-Carranza et al., 2012; Liljegren, 2012; Aalen et al., 2013; Kim, 2014; Tucker and Kim, 2015). It is possible that, even if IDA-signaling plays a more prominent role in regulating the synthesis of a boundary layer, a separation phenotype in *IDA*-like suppressed mutants will not be obvious in many abscission systems because disassembly of the cell wall and middle lamella is sufficient when the distal organ has a mass greater than that of a flower petal, stamen, or sepal. Nevertheless, we propose a modification to the abscission model to include synthesis of an extensible boundary layer early in the abscission process that is different from the deposition of a protective layer (Figure 4). However, the waxy cuticle synthesized early during separation may become a part of a more rigid protective layer deposited after separation. Moreover, as suggested much earlier by Patterson and Bleecker (2004), we reassert that multiple hormonal and peptide signals regulate the rate of abscission and successful organ separation and these different signals do not necessarily regulate the same set of genes. Of interest in this regard was that differential gene expression of ethylene (e.g., ERFs) and auxin-associated genes (e.g., SAUR, AUX.IAA) was not greatly changed between WT and *hae-hsl2* Arabidopsis receptacles at flower stage 15 (Figures 2C, 3C). This further supports a model where IDA signaling does not directly affect ethylene or auxin signaling in Arabidopsis floral organ abscission and that multiple signals influence abscission. What is necessary is discovering how these signals work together and independently to bring about a successful separation process.

## Concluding remarks

Fruit softening includes expression of cell wall and middle lamella degrading enzymes (Tucker, 2014). The fruit ripening genes that affect cell wall modifications are not necessarily the same genes as those expressed in abscission, but they likely have similar functions. However, cell separation is not the same in a ripe fruit as it is in the fracture plane. Evaluation of the transcriptome of the pericarp of a wild-type ripening tomato (Osorio et al., 2011) indicated strong up-regulation of *CELs, PGs, EXPs*, and *XTHs* but not a marked increase in wax synthesis genes or lipid transfer proteins (Supplemental File S6). Could it be that an important difference between fruit ripening and abscission is the synthesis of a cuticle-like boundary on the abscission cells? Our proposal that a waxy-like cuticle is important to the abscission process is further supported by observations for separation during organogenesis. It seems to make evolutionary sense that abscission of plant organs would be an adaptation of a primal process occurring in the meristem. Although we do not know the exact composition of the abscission boundary layer, our proposal can be tested genetically and biochemically.

## Sequence submission

The raw soybean sequence files have been submitted to the National Center for Biotechnology Information (NCBI) SRA databases with the study accession SRP050050. The soybean results including RPKM and annotations for all 54,175 genes can be downloaded at http://sgil.ba.ars.usda.gov/mtucker/Public/Tucker.html. The raw tomato sequence data and array information were submitted to the Gene Expression Omnibus (GEO) at the NCBI with GEO IDs GSE45355 and GSE45356, and array ID AMADID:043310.

## Author contributions

Conceived, designed experiments and interpreted data: JK and MT. Performed experiments and prepared and analyzed data: SS and RY. Analyzed and interpreted data: SH and SM.

## Funding

This work was supported by a United States - Israel Binational Agricultural and Development Fund (BARD) US-4571-12C grant to MT, SM, and SP.

### Conflict of interest statement

The authors declare that the research was conducted in the absence of any commercial or financial relationships that could be construed as a potential conflict of interest.

## Supplementary material

Supplemental Figure S1 **Soybean explant preparation**.

Supplemental File S2 **Excel file for gene names, expression, annotation and graphs for all the soybean genes included in Figures 2A, 3A**.

Supplemental File S3 **Excel file for gene names, expression, annotation and graphs for all the tomato genes included in Figures 2B, 3B**.

Supplemental File S4 **Excel file for gene names, expression, annotation and graphs for all the ***Arabidopsis*** genes included in Figures 2C, 3C**.

Supplemental File S5 **Phylogenetic tree of dicot, monocot, pine and moss PAR1-like protein sequences. Alignment was performed with the signal peptides removed**.

Supplemental File S6 **Excel file for gene names, expression and annotation for tomato fruit genes**.

## References

[B1] Aalen R. B. Wildhagen M. Stø I. M. Butenko M. A. (2013). IDA: A peptide ligand regulating cell separation processes in *Arabidopsis*. J. Exp. Bot. 64, 5253–5261. 10.1093/jxb/ert338 24151306

[B2] Abeles F. B. Morgan P. W. Salveit M. E. (1992). Ethylene in Plant Biology. New York, NY: Academic Press.

[B3] Addicott F. T. (1982). Abscission. Berkely, CA: California University Press.

[B4] Agusti J. Merelo P. Cercós M. Tadeo F. R. Talón M. (2009). Comparative transcriptional survey between laser-microdissected cells from laminar abscission zone and petiolar cortical tissue during ethylene-promoted abscission in citrus leaves. BMC Plant Biol. 9:127. 10.1186/1471-2229-9-127 19852773 PMC2770498

[B5] Almagro L. Gómez Ros L. V. Belchi-Navarro S. Bru R. Ros Barceló A. Pedreño M. A. (2009). Class III peroxidases in plant defence reactions. J. Exp. Bot. 60, 377–390. 10.1093/jxb/ern277 19073963

[B6] Basu M. M. González-Carranza Z. H. Azam-Ali S. Tang S. Shahid A. A. Roberts J. A. (2013). The manipulation of auxin in the abscission zone cells of *Arabidopsis* flowers reveals that indoleacetic acid signaling is a prerequisite for organ shedding. Plant Physiol. 162, 96–106. 10.1104/pp.113.216234 23509178 PMC3641234

[B7] Bleecker A. B. Patterson S. E. (1997). Last exit: Senescence, abscission, and meristem arrest in Arabidopsis. Plant Cell 9, 1169–1179. 10.1105/tpc.9.7.1169 9254934 PMC156989

[B8] Butenko M. A. Patterson S. E. Grini P. E. Stenvik G. E. Amundsen S. S. Mandal A.. (2003). *Inflorescence deficient in abscission* controls floral organ abscission in Arabidopsis and identifies a novel family of putative ligands in plants. Plant Cell 15, 2296–2307. 10.1105/tpc.014365 12972671 PMC197296

[B9] Butenko M. A. Wildhagen M. Albert M. Jehle A. Kalbacher H. Aalen R. B.. (2014). Tools and strategies to match peptide-ligand receptor pairs. Plant Cell 26, 1838–1847. 10.1105/tpc.113.120071 24808051 PMC4079353

[B10] Cai S. Lashbrook C. C. (2008). Stamen abscission zone transcriptome profiling reveals new candidates for abscission control: Enhanced retention of floral organs in transgenic plants overexpressing Arabidopsis ZINC FINGER PROTEIN2. Plant Physiol. 146, 1305–1321. 10.1104/pp.107.110908 18192438 PMC2259061

[B11] Carpita N. C. Gibeaut D. M. (1993). Structural models of primary cell walls in flowering plants: Consistency of molecular structure with the physical properties of the walls during growth. Plant J. 3, 1–30. 10.1111/j.1365-313X.1993.tb00007.x 8401598

[B12] Cho S. K. Larue C. T. Chevalier D. Wang H. Jinn T. L. Zhang S.. (2008). Regulation of floral organ abscission in *Arabidopsis thaliana*. Proc. Natl. Acad. Sci. U.S.A. 105, 15629–15634. 10.1073/pnas.0805539105 18809915 PMC2563077

[B13] Cosgrove D. J. (2001). Wall structure and wall loosening. A look backwards and forwards. Plant Physiol. 125, 131–134. 10.1104/pp.125.1.131 11154315 PMC1539344

[B14] Craker L. E. Abeles F. B. (1969). Abscission: Quantitative measurement with a recording abscissor. Plant Physiol. 44, 1139–1143. 10.1104/pp.44.8.1139 16657180 PMC396229

[B15] Del Campillo E. Bennett A. B. (1996). Pedicel breakstrength and cellulase gene expression during tomato flower abscission. Plant Physiol. 111, 813–820. 10.1104/pp.111.3.813 8754682 PMC157899

[B16] Del Campillo E. Lewis L. N. (1992). Identification and kinetics of accumulation of proteins induced by ethylene in bean abscission zones. Plant Physiol. 98, 955–961. 10.1104/pp.98.3.955 16668770 PMC1080293

[B17] Doblin M. S. Kurek I. Jacob-Wilk D. Delmer D. P. (2002). Cellulose biosynthesis in plants: From genes to rosettes. Plant Cell Physiol. 43, 1407–1420. 10.1093/pcp/pcf164 12514238

[B18] Eklöf J. M. Ruda M. C. Brumer H. (2012). Distinguishing xyloglucanase activity in endo-β(1 → 4)glucanases. Methods Enzymol. 510, 97–120. 10.1016/B978-0-12-415931-0.00006-9 22608723

[B19] Estornell L. H. Agustí J. Merelo P. Talón M. Tadeo F. R. (2013). Elucidating mechanisms underlying organ abscission. Plant Sci. 199–200, 48–60. 10.1016/j.plantsci.2012.10.008 23265318

[B20] González-Carranza Z. H. Elliott K. A. Roberts J. A. (2007). Expression of polygalacturonases and evidence to support their role during cell separation processes in *Arabidopsis thaliana*. J. Exp. Bot. 58, 3719–3730. 10.1093/jxb/erm222 17928369

[B21] González-Carranza Z. H. Shahid A. A. Zhang L. Liu Y. Ninsuwan U. Roberts J. A. (2012). A novel approach to dissect the abscission process in Arabidopsis. Plant Physiol. 160, 1342–1356. 10.1104/pp.112.205955 22992509 PMC3490599

[B22] Grassi S. Piro G. Lee J. M. Zheng Y. Fei Z. Dalessandro G.. (2013). Comparative genomics reveals candidate carotenoid pathway regulators of ripening watermelon fruit. BMC Genomics 14:781. 10.1186/1471-2164-14-781 24219562 PMC3840736

[B23] Hall J. L. Sexton R. (1974). Fine structure and cytochemistry of the abscission zone cells of phaseolus leaves II. Localization of peroxidase and acid phosphatase in the separation zone cells. Ann. Bot. 38, 855–858.

[B24] Hamann T. (2012). Plant cell wall integrity maintenance as an essential component of biotic stress response mechanisms. Front. Plant Sci. 3:77. 10.3389/fpls.2012.00077 22629279 PMC3355559

[B25] Hayashi T. Kaida R. (2011). Functions of xyloglucan in plant cells. Mol. Plant 4, 17–24. 10.1093/mp/ssq063 20943810

[B26] Herbers K. Mönke G. Badur R. Sonnewald U. (1995). A simplified procedure for the subtractive cDNA cloning of photoassimilate-responding genes: Isolation of cDNAs encoding a new class of pathogenesis-related proteins. Plant Mol. Biol. 29, 1027–1038. 10.1007/BF00014975 8555446 PMC7088993

[B27] Kalaitzis P. Solomos T. Tucker M. L. (1997). Three different polygalacturonases are expressed in tomato leaf and flower abscission, each with a different temporal expression pattern. Plant Physiol. 113, 1303–1308. 10.1104/pp.113.4.1303 9112778 PMC158253

[B28] Kim J. (2014). Four shades of detachment: Regulation of floral organ abscission. Plant Signal Behav. 9:e976154. 10.4161/15592324.2014.976154 25482787 PMC4623469

[B29] Kim J. Dotson B. Rey C. Lindsey J. Bleecker A. B. Binder B. M.. (2013). New clothes for the jasmonic acid receptor *COI1*: Delayed abscission, meristem arrest and apical dominance. PLoS ONE 8:e60505. 10.1371/journal.pone.0060505 23573263 PMC3613422

[B30] Kim J. Patterson S. E. (2006). Expression divergence and functional redundancy of polygalacturonases in floral organ abscission. Plant Signal Behav. 1, 281–283. 10.4161/psb.1.6.3541 19704626 PMC2634239

[B31] Lanahan M. B. Yen H. C. Giovannoni J. J. Klee H. J. (1994). The *never ripe* mutation blocks ethylene perception in tomato. Plant Cell 6, 521–530. 10.1105/tpc.6.4.521 8205003 PMC160455

[B32] Langmead B. Trapnell C. Pop M. Salzberg S. L. (2009). Ultrafast and memory-efficient alignment of short DNA sequences to the human genome. Genome Biol. 10:R25. 10.1186/gb-2009-10-3-r25 19261174 PMC2690996

[B33] Lashbrook C. C. Cai S. (2008). Cell wall remodeling in Arabidopsis stamen abscission zones: Temporal aspects of control inferred from transcriptional profiling. Plant Signal Behav. 3, 733–736. 10.4161/psb.3.9.6489 19704843 PMC2634574

[B34] Liljegren S. J. (2012). Organ abscission: Exit strategies require signals and moving traffic. Curr. Opin. Plant Biol. 15, 670–676. 10.1016/j.pbi.2012.09.012 23047135

[B35] Liu B. Butenko M. A. Shi C. L. Bolivar J. L. Winge P. Stenvik G. E.. (2013). NEVERSHED and INFLORESCENCE DEFICIENT IN ABSCISSION are differentially required for cell expansion and cell separation during floral organ abscission in *Arabidopsis thaliana*. J. Exp. Bot. 64, 5345–5357. 10.1093/jxb/ert232 23963677

[B36] Matheis G. Whitaker J. R. (1984). Peroxidase-catalyzed cross linking of proteins. J. Protein Chem. 3, 35–48. 10.1007/BF01024835

[B37] Meir S. Philosoph-Hadas S. Sundaresan S. Selvaraj K. S. Burd S. Ophir R.. (2010). Microarray analysis of the abscission-related transcriptome in the tomato flower abscission zone in response to auxin depletion. Plant Physiol. 154, 1929–1956. 10.1104/pp.110.160697 20947671 PMC2996037

[B38] Meir S. Philosoph-Hadas S. Sundaresan S. Selvaraj K. S. Burd S. Ophir R.. (2011). Identification of defense-related genes newly-associated with tomato flower abscission. Plant Signal Behav. 6, 590–593. 10.4161/psb.6.4.15043 21543890 PMC3142400

[B39] Mortazavi A. Williams B. A. McCue K. Schaeffer L. Wold B. (2008). Mapping and quantifying mammalian transcriptomes by RNA-Seq. Nat. Methods 5, 621–628. 10.1038/nmeth.1226 18516045 PMC13303166

[B40] Nakano T. Fujisawa M. Shima Y. Ito Y. (2013). Expression profiling of tomato pre-abscission pedicels provides insights into abscission zone properties including competence to respond to abscission signals. BMC Plant Biol. 13:40. 10.1186/1471-2229-13-40 23497084 PMC3600680

[B41] Niederhuth C. E. Patharkar O. R. Walker J. C. (2013). Transcriptional profiling of the Arabidopsis abscission mutant *hae hsl2* by RNA-seq. BMC Genomics 14:37. 10.1186/1471-2164-14-37 23327667 PMC3566969

[B42] Osorio S. Alba R. Damasceno C. M. Lopez-Casado G. Lohse M. Zanor M. I.. (2011). Systems biology of tomato fruit development: Combined transcript, protein, and metabolite analysis of tomato transcription factor (nor, rin) and ethylene receptor (Nr) mutants reveals novel regulatory interactions. Plant Physiol. 157, 405–425. 10.1104/pp.111.175463 21795583 PMC3165888

[B43] Patterson S. E. (2001). Cutting loose. Abscission and dehiscence in Arabidopsis. Plant Physiol. 126, 494–500. 10.1104/pp.126.2.494 11402180 PMC1540116

[B44] Patterson S. E. Bleecker A. B. (2004). Ethylene-dependent and -independent processes associated with floral organ abscission in Arabidopsis. Plant Physiol. 134, 194–203. 10.1104/pp.103.028027 14701913 PMC316299

[B45] Peaucelle A. Braybrook S. A. Le Guillou L. Bron E. Kuhlemeier C. Höfte H. (2011). Pectin-induced changes in cell wall mechanics underlie organ initiation in Arabidopsis. Curr. Biol. 21, 1720–1726. 10.1016/j.cub.2011.08.057 21982593

[B46] Roberts J. A. Elliott K. A. Gonzalez-Carranza Z. H. (2002). Abscission, dehiscence, and other cell separation processes. Annu. Rev. Plant Biol. 53, 131–158. 10.1146/annurev.arplant.53.092701.180236 12221970

[B47] Rowland O. Zheng H. Hepworth S. R. Lam P. Jetter R. Kunst L. (2006). CER4 encodes an alcohol-forming fatty acyl-coenzyme A reductase involved in cuticular wax production in Arabidopsis. Plant Physiol. 142, 866–877. 10.1104/pp.106.086785 16980563 PMC1630741

[B48] Samuels L. Kunst L. Jetter R. (2008). Sealing plant surfaces: Cuticular wax formation by epidermal cells. Annu. Rev. Plant Biol. 59, 683–707. 10.1146/annurev.arplant.59.103006.093219 18251711

[B49] Sexton R. Roberts J. A. (1982). Cell biology of abscission. Annu. Rev. Plant Physiol. 33, 133–162. 10.1146/annurev.pp.33.060182.001025

[B50] Shi J. X. Malitsky S. De Oliveira S. Branigan C. Franke R. B. Schreiber L.. (2011). SHINE transcription factors act redundantly to pattern the archetypal surface of Arabidopsis flower organs. PLoS Genet. 7:e1001388. 10.1371/journal.pgen.1001388 21637781 PMC3102738

[B51] Smirnova A. Leide J. Riederer M. (2013). Deficiency in a very-long-chain fatty acid beta-ketoacyl-coenzyme a synthase of tomato impairs microgametogenesis and causes floral organ fusion. Plant Physiol. 161, 196–209. 10.1104/pp.112.206656 23144186 PMC3532251

[B52] Soler M. Serra O. Molinas M. Huguet G. Fluch S. Figueras M. (2007). A genomic approach to suberin biosynthesis and cork differentiation. Plant Physiol. 144, 419–431. 10.1104/pp.106.094227 17351057 PMC1913797

[B53] Stenvik G. E. Butenko M. A. Aalen R. B. (2008). Identification of a putative receptor-ligand pair controlling cell separation in plants. Plant Signal Behav. 3, 1109–1110. 10.4161/psb.3.12.7009 19704449 PMC2634470

[B54] Suh M. C. Samuels A. L. Jetter R. Kunst L. Pollard M. Ohlrogge J.. (2005). Cuticular lipid composition, surface structure, and gene expression in Arabidopsis stem epidermis. Plant Physiol. 139, 1649–1665. 10.1104/pp.105.070805 16299169 PMC1310549

[B55] Taylor J. E. Whitelaw C. A. (2001). Signals in abscission. New Phytol. 151, 323–339. 10.1046/j.0028-646x.2001.00194.x

[B56] Trapnell C. Pachter L. Salzberg S. L. (2009). TopHat: Discovering splice junctions with RNA-Seq. Bioinformatics 25, 1105–1111. 10.1093/bioinformatics/btp120 19289445 PMC2672628

[B57] Tucker M. L. (2014). Cell-wall metabolism and softening during ripening, in Fruit Ripening: Physiology, Signalling and Genomics, eds Nath P. Bouzayen M. Mattoo A.K. Pech J. C. (Wallingford, CT: CABI), 48–62.

[B58] Tucker M. L. Burke A. Murphy C. A. Thai V. K. Ehrenfried M. L. (2007). Gene expression profiles for cell wall-modifying proteins associated with soybean cyst nematode infection, petiole abscission, root tips, flowers, apical buds, and leaves. J. Exp. Bot. 58, 3395–3406. 10.1093/jxb/erm188 17916637

[B59] Tucker M. L. Kim J. (2015). Abscission research: What we know and what we still need to study. Stewart Posthar. Rev. 11, 1–7. 10.2212/spr.2015.2.1

[B60] Tucker M. L. Sexton R. Del Campillo E. Lewis L. N. (1988). Bean abscission cellulase: Characterization of a cDNA clone and regulation of gene expression by ethylene and auxin. Plant Physiol. 88, 1257–1262. 10.1104/pp.88.4.1257 16666452 PMC1055750

[B61] Tucker M. L. Yang R. (2012). IDA-like gene expression in soybean and tomato leaf abscission and requirement for a diffusible stelar abscission signal. AoB Plants 2012:pls035. 10.1093/aobpla/pls035 23585923 PMC3624929

[B62] Urbanowicz B. R. Bennett A. B. Del Campillo E. Catalá C. Hayashi T. Henrissat B.. (2007). Structural organization and a standardized nomenclature for plant endo-1,4-β-glucanases (cellulases) of glycosyl hydrolase family 9. Plant Physiol. 144, 1693–1696. 10.1104/pp.107.102574 17687051 PMC1949884

[B63] Vie A. K. Najafi J. Liu B. Winge P. Butenko M. A. Hornslien K. S.. (2015). The *IDA/IDA-LIKE* and *PIP/PIP-LIKE* gene families in *Arabidopsis*: phylogenetic relationship, expression patterns, and transcriptional effect of the PIPL3 peptide. J. Exp. Bot. 66, 5351–5365. 10.1093/jxb/erv285 26062745 PMC4526919

[B64] Voigt C. A. (2014). Callose-mediated resistance to pathogenic intruders in plant defense-related papillae. Front. Plant Sci. 5:168. 10.3389/fpls.2014.00168 24808903 PMC4009422

[B65] Wang X. Liu D. Li A. Sun X. Zhang R. Wu L.. (2013). Transcriptome analysis of tomato flower pedicel tissues reveals abscission zone-specific modulation of key meristem activity genes. PLoS ONE 8:e55238. 10.1371/journal.pone.0055238 23390523 PMC3563536

[B66] Yeats T. H. Rose J. K. (2013). The formation and function of plant cuticles. Plant Physiol. 163, 5–20. 10.1104/pp.113.222737 23893170 PMC3762664

[B67] Žádníková P. Simon R. (2014). How boundaries control plant development. Curr. Opin. Plant Biol. 17, 116–125. 10.1016/j.pbi.2013.11.013 24507503

[B68] Zhong S. Joung J. G. Zheng Y. Chen Y. R. Liu B. Shao Y.. (2011). High-throughput illumina strand-specific RNA sequencing library preparation. Cold Spring Harb. Protoc. 2011, 940–949. 10.1101/pdb.prot5652 21807852

