# Supplementary figures and images for: Examination of the Abscission-Associated Transcriptomes for Soybean, Tomato, and Arabidopsis Highlights the Conserved Biosynthesis of an Extensible Extracellular Matrix and Boundary Layer
Source: Front Plant Sci. 2015 Dec 15;6:1109. doi: 10.3389/fpls.2015.01109 (PMC4678212; doi:10.3389/fpls.2015.01109)

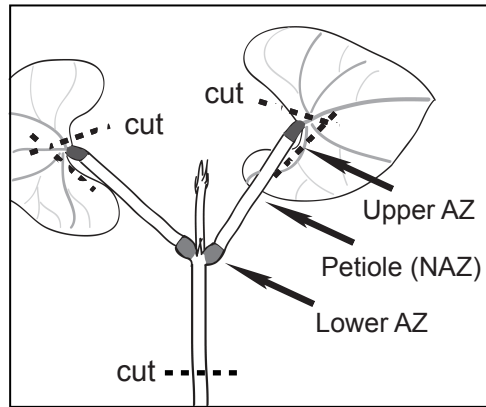

**Supplemental Figure S1: Soybean explant preparation.**

Supplement: Supplemental Figure S1 — Soybean explant preparation. [file Image1.PDF]
