# Supplementary material for: Examination of the Abscission-Associated Transcriptomes for Soybean, Tomato, and Arabidopsis Highlights the Conserved Biosynthesis of an Extensible Extracellular Matrix and Boundary Layer
Source: Front Plant Sci. 2015 Dec 15;6:1109. doi: 10.3389/fpls.2015.01109 (PMC4678212; doi:10.3389/fpls.2015.01109)
Supplement: Supplemental File S5 — Phylogenetic tree of dicot, monocot, pine and moss PAR1-like protein sequences. Alignment was performed with the signal peptides removed. [file Image2.PDF]

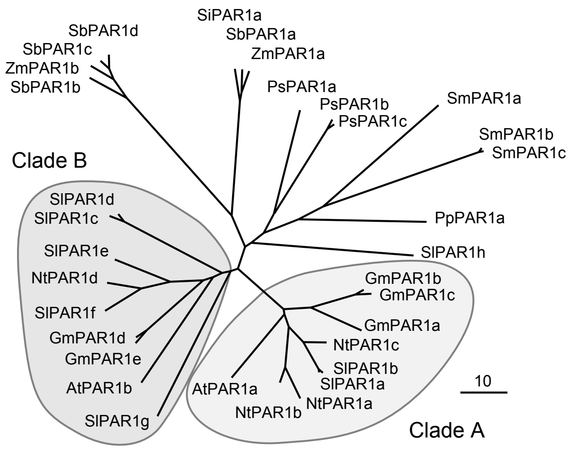

Figure S4. Phylogenetic tree of selected PAR1-like protein sequences in plants. Alignment was performed with the signal peptides removed. The names correspond to sequence IDs listed in **Table S4**.

Table S4. Naming and properties of PAR1-like genes: *Nicotiana tabacum* (tobacco), *Arabidopsis thaliana* (Arabidopsis), *Glycine max* (soybean), *Solanum lycopersicum* (tomato), *Zea mays* (maize), *Sorghum bicolor* (sorghum), *Setaria italica* (foxtail millet), *Picea sitchensis* (Sitka spruce), *Selaginella moellendorffii* (club moss) and *Physcomitrella patens* (moss).

Gene Name	Accession/ID	Mol Wt no SP [1] (kDa)	Percent aa Similarity [2] no SP [1]
NtPAR1a	X83853	17.4	100
NtPAR1b	X83851	17.5	94
NtPAR1c	X83852	17.1	89
NtPAR1d	AB041515	16.2	62
AtPAR1a	AT5G52390	18.6	73
AtPAR1b	AT3G54040	16.7	60
GmPAR1a	Glyma09g25420.1	18.9	75
GmPAR1b	Glyma10g37940.1	17.8	83
GmPAR1c	Glyma20g29860.1	18.0	81
GmPAR1d	Glyma01g26840.2	16.7	68
GmPAR1e	Glyma03g15130.1	17.0	68
SlPAR1a	Solyc03g025670.2.1	17.4	87
SlPAR1b	Solyc03g025680.2.1	17.5	87
SlPAR1c	Solyc09g011790.2.1	16.3	60
SlPAR1d	Solyc09g011800.2.1	16.2	59
SlPAR1e	Solyc10g085010.1.1	17.2	61
SlPAR1f	Solyc10g086710.1.1	16.2	62
SlPAR1g	Solyc06g005920.1.1	18.3	60
SlPAR1h	Solyc01g099920.2.1	22.6	46
ZmPAR1a	AFW77700	15.8	60
ZmPAR1a	AFW78200	15.2	52
SbPAR1a	XM_002439588	16.2	58
SbPAR1b	XM_002439851	17.8	38
SbPAR1c	XM_002439852	15.3	50
SbPAR1d	XM_002439853	15.3	49
SiPAR1a	XP_004962356	16.8	59
PsPAR1a	ABK22647	18.1	59
PsPAR1b	ABK26953	19.7	63
PsPAR1c	ABK26944	19.8	62
SmPAR1a	XP_002971544	19.3	51
SmPAR1b	XP_002971753	18.4	52
SmPAR1c	XP_002993093	18.4	52
PpPAR1a	Pp1s185_4V6	30.3	36

[1] no SP – signal peptide identified by SignalP 4.0 is removed.
[2] Percent amino acid similarity using MacVector 12.7, ClustalW.
